# Supplementary material for: Trichoderma harzianum Cellobiohydrolase Thph2 Induces Reactive Oxygen Species-Mediated Resistance Against Southern Corn Leaf Blight in Maize
Source: J Fungi (Basel). 2025 Aug 27;11(9):629. doi: 10.3390/jof11090629 (PMC12470592; doi:10.3390/jof11090629)
Supplement: Supplementary file 1 [file jof-11-00629-s001.zip › jof-3758312-supplementary.pdf]

**Table S1.** Primers used in this study.

| Primer name | Sequence (5'-3'; restriction sites underlined) | Description |
|---|---|---|
| | | |
| 18s-F | CCTGCGGCTTAATTGACTC | qRT-PCR primer for 18s as reference |
| 18s-R | GTTAGCAGGCTGAGGTCTCG | |
| AOS-F | TTCCTCCGCTACGACACATT | qRT-PCR primer for *ZmAOS* |
| AOS-R | CCGAGGGTGAGAGAGAAGTC | |
| HPL-F | CCACACACGGGAGATAGCTA | qRT-PCR primer for *ZmHPL* |
| HPL-R | GAAGAACTCGTCCTGGCTCT | |
| LOX5-F | TCGCGTCTACCGTTACGACTACTA | qRT-PCR primer for *LOX5* |
| LOX5-R | TTCAGGTTCAGCAGGAAAAGC | |
| OPR1-F | CGTATGGGAGGCTGTTCTTG | qRT-PCR primer for *OPR1* |
| OPR1-R | AGCGGTCGTATTTGTTGAGTG | |
| ZmPR1-F | GGCGAGAGCCCCTACTAGAC | qRT-PCR primer for *ZmPR1* |
| ZmPR1-R | AAATCGCCTGCATGGTTTTA | |
| ZmPR5-F | GTCATCGACGGCTACAACCT | qRT-PCR primer for *ZmPR5* |
| ZmPR5-R | CACGGGCAGAAGGTGACT | |
| PAL-F | GAAGCTCATGTCGTCCACCTA | qRT-PCR primer for *PAL* |
| PAL-R | GTTCATGGTCAGCACCTTCTT | |
